# A Genetic Algorithm for the Bi-Level Topological Design of Local Area Networks

**DOI:** 10.1371/journal.pone.0128067

**Published:** 2015-06-23

**Authors:** José-Fernando Camacho-Vallejo, Julio Mar-Ortiz, Francisco López-Ramos, Ricardo Pedraza Rodríguez

**Affiliations:** 1 Universidad Autónoma de Nuevo León, Facultad de Ciencias Físico-Matemáticas, San Nicolás de los Garza, Nuevo León, México; 2 Universidad Autónoma de Tamaulipas, Facultad de Ingeniería, Tampico, Tamaulipas, México; 3 School of Transport Engineering, Pontificia Universidad Católica de Valparaíso, Valparaiso, Chile; University of Amsterdam, NETHERLANDS

## Abstract

Local access networks (LAN) are commonly used as communication infrastructures which meet the demand of a set of users in the local environment. Usually these networks consist of several LAN segments connected by bridges. The topological LAN design bi-level problem consists on assigning users to clusters and the union of clusters by bridges in order to obtain a minimum response time network with minimum connection cost. Therefore, the decision of optimally assigning users to clusters will be made by the leader and the follower will make the decision of connecting all the clusters while forming a spanning tree. In this paper, we propose a genetic algorithm for solving the bi-level topological design of a Local Access Network. Our solution method considers the Stackelberg equilibrium to solve the bi-level problem. The Stackelberg-Genetic algorithm procedure deals with the fact that the follower’s problem cannot be optimally solved in a straightforward manner. The computational results obtained from two different sets of instances show that the performance of the developed algorithm is efficient and that it is more suitable for solving the bi-level problem than a previous Nash-Genetic approach.

## Introduction

The design of computer and telecommunication networks is a hard constrained combinatorial optimization problem that has received considerable attention from practitioners and researchers during the recent years. The telecommunication network design problem consists of deciding the number, types, and locations of the network active elements (hubs, switches, and routers), as well as the links and their capacities. Several conflicting and hierarchical objectives such as monetary cost, network delay, and maximum number of hubs have to be optimized to achieve a desirable solution. In the existing literature we can identify two kinds of problems related to the telecommunication networks design: hub location [1] and topological design [2]. The hub location problem is concerned with locating hub facilities in a network and allocating demand nodes to hubs in order to route the traffic between origin–destination pairs. The problem of designing the networks topology consists on the selection of a subset of links which optimize a predetermined performance criterion. In this paper we focus on a topological design of local area networks (LANs).

LANs are commonly used as communication infrastructures which meet the demand for a set of users in the local environment. Usually these networks consist of several LAN segments connected by bridges. The topological design problem of a LAN consists on finding the best configuration between users and clusters that optimizes a defined (or several) performance criterion, such as, equipment costs, connection costs, response times, network reliability, among others (see [3]). These performance criteria are very important and are significantly affected by the network topology. The problem of optimal network design is a hard combinatorial problem involving assignment and routing decisions. The assignment problem is to determine the best way to assign users to clusters, while the routing problem determines the segments where the clusters need to be interconnected through bridges leading us to a spanning tree. Typically the design of communication networks requires the existence of a spanning tree, in which each node must be able to communicate with every other node. However, [4] remarks that spanning trees solutions do not provide a reliable design but a minimum cost design.

The LAN topology design has been a very active research area in the last two decades. Many authors have proposed both exact methods (e.g. [5], [6] and [7]) and heuristics ([8], [9], [10], [4] and [11]) for the design of LANs and its variants, where genetic algorithms have had a strong preference over other meta-heuristics. One of the first descriptions of the operation and structure of LANs is found in [12]. Particularly relevant to our research are [13] and [14]. The former research proposes a model of nonlinear integer programming which minimizes the average delay in the network as performance criteria and applies a genetic algorithm to solve it. Starting with the approach presented in [13], in [14] a bi-level model is proposed and a Genetic algorithm based on Nash-equilibrium to solve the problem is designed.

In this paper, a genetic algorithm that considers the Stackelberg equilibrium is proposed as a solution method for the bi-level topological design of a Local Area Network. The Stackelberg-Genetic procedure assumes that the follower rational reacts to a leader’s decision; this is, an acceptable spanning tree is selected by the follower due to the difficulty of finding its optimal response in an efficient way. This paper has two objectives: the primary objective is to investigate the performance of the Stackelberg-Genetic algorithm for solving the bi-level problem, and the secondary objective is to show the difference in solving bi-level problems with either Stackelberg or Nash approaches. The remainder of this section describes the previous contributions consisting in metaheuristics developed for this and related bi-level problems. Section 2 presents the bi-level mathematical model. Section 3 is devoted to describe the solution method proposed for obtaining high-quality bi-level solutions. Section 4 shows the computational experiments carried out on previously reported instances and in new generated ones. Also, in this section a Nash-Genetic algorithm similar to the one described in [14] is used for solving the benchmark instances and the obtained results are discussed. The paper is finished with the associated conclusions remarking the importance of solving the bi-level problems with the appropriate methodology.

### Related literature

In the last 25 years the field of bi-level optimization has received considerable attention reflected in a wide variety of applications, where metaheuristic algorithms have been considered as a good alternative for finding high quality solutions to considerable size bi-level problems. There are papers in the fields of environmental studies (see [15]), humanitarian logistics (see [16] and [17]), network design (see [18] and [19]), transportation (see [20] and [21]), toll setting (see [22] and [23]), location (see [24] and [25]), production planning (see [26]), among many others. For a description of more applications and solution methods we refer the reader to [27] and [28]. Table 1 summarizes some relevant previous works ([29–38]) on metaheuristics designed for solving bi-level problems. The main objective here is describing the interaction strategy between leader and follower. Basically, three interaction strategies are identified: *A*) for each leader decision it is necessary to solve the lower level problem, *B*) the lower level problem is solved after a predetermined number of iterations, and *C*) both leader's and follower's populations cooperate or coevolves after a predefined number of iterations.

**Table 1:** Relevant previous work.

Reference	Type	Description
Mathieu et al. [29]	*A*	To the best of our knowledge this is the first application in the literature of genetic algorithms for solving bi-level programming problems. In this paper, the initial population of solutions was leader solutions and the follower responses were obtained by directly optimizing the linear lower level problem. For each leader’s solution the follower’s problem is solved exactly.
Oduguwa and Roy [30]	*C*	Propose a general scheme for a genetic algorithm capable of solving different applications of bi-level programming problems. It is assumed that the follower cooperates with the leader when the former obtains the lower level optimal response. The cooperation is made at the end of each iteration when a synchronization (interchange) between leader's and follower's populations is conducted in order to preserve the interactive nature of the problem.
Bhadury et al. [31]	*A*	Several variations of genetic algorithms are proposed to solve a wide variety of location problems, including competitive location problems. In order to solve the lower level problem they designed a greedy heuristic, which was implemented every time a new leader's solution was generated.
Yang et al. [32]	*A*	Propose a hybrid algorithm that combines the Simplex method, genetic algorithms, stochastic and fuzzy simulations, to solve a bi-level location problem where the flow from the lower level to the upper level is stochastic. For each leader’s solution the lower level is solved exactly.
Aleekseva et al. [33]	*A*	Propose a memetic hybrid algorithm that combines the principles of evolutionary algorithms with tabu search for a competitive *p-Median* problem. For each leader evaluation they solve the lower level problem through a commercial software.
Vasilyev and Klimentova [34]	*A*	Design a hybrid algorithm that combines the simulated annealing method with the branch-and-cut method, in order to obtain upper bounds for the facility location problem with users’ preferences. During the simulated annealing method they solved the lower level problem for each leader’s solution.
Gallo et al. [21]	*B*	Propose a scatter search algorithm to solve urban transportation network design problem. The upper level can be seen as a model for solving the topological network design problem. The lower level model aims to solve the signal setting problem. In order to avoid the necessity of solving the lower level at each iteration they propose to solve the signal setting problem with a local approach. Accordingly, the signal setting problem was formulated as an asymmetric equilibrium assignment problem, where only the topological variables assume the role of decision variables, and both, the signal settings and the equilibrium traffic flows, are descriptive variables reducing the bi-level problem into a single-level one. Even though, due to the size of the neighborhoods they use a random search method for improving the solutions
Kücükaydin et al. [35]	*A*	Design a tabu search algorithm to solve a competitive facility location bi-level problem. Both, the leader and follower, seek to maximize their own profit considering the already existent facilities and the new ones. The lower level is solved by a branch-and-bound algorithm with a non-linear programming relaxation due to propositions introduced by them.
Calvete et al. [26]	*A*	Propose an ant colony optimization based algorithm to solve a bi-level production-distribution planning problem, where the upper level consists on a multi-depot vehicle routing problem, and the lower level model aims to solve the problem of minimizing manufacturing costs. In the bi-level ant colony optimization algorithm they exactly solved the lower level problem.
Legillon et al. [36]	*C*	Design a co-evolutionary algorithm to solve a bi-level production and distribution planning problem. They propose two initial populations, one for the leader and one for the follower which periodically exchange information to improve the individuals. The individuals are created by the union of leader-follower solutions. The fitness function is evaluated based on the leaders’ objective function.
Brotcorne et al. [37]	*A*	Design a tabu search algorithm to solve the bi-level toll setting problem in a transportation network. The leader wants to maximize the profit obtained from the tolls from a transportation network, while the follower seeks to minimize their total travel cost. In order to obtain the followers’ optimal response they consider a lower level reformulation, then apply column generation and solved the resulting problem by inverse optimization.
Camacho et al. [38]	*A*	Propose a Stackelberg-Evolutionary algorithm to solve a facility location bi-level problem with customers’ preferences. The upper level seeks to minimize the location and distribution costs and the follower tries to minimize a utility function based on the preferences. At each iteration of the proposed algorithm a leader's solution is obtained, then it is provided to the follower which directly optimizes the lower level allocation problem in order to obtain its optimal response, finally the upper level objective function is evaluated.

In Table 1, it can be observed that in the majority of the previous works that considered metaheuristics for solving bi-level optimization problems; the follower’s problem is exactly solved, i.e. an *A* appears in the second column. The main difficulty arising from the problem considered in this paper is that the follower’s problem is a hard combinatorial problem. Hence, we are not able to solve it optimally in a reasonable computational time due to the high number of times that the follower’s problem needs to be solved. This is where the rational reaction of the follower takes place in sense of not optimally solving its problem and conform its response to a good quality solution. This fact is described in subsection 3.1.

Moreover, from a game theory point of view, two-player problems may be approached by either Nash (see [39]) or Stackelberg (see [40]) frameworks. Both approaches have been widely studied in the literature. The kind of leader-follower problem resembles the Stackelberg games. A Stackelberg game is composed of an upper-level vector of decision variables *y* for the leader, and a lower-level vector of decision variables *x* for the follower. It is assumed that the leader is given the first choice and selects a solution *y* in accordance with his constraints in order to optimize his objective function; this decision is made while taking into account the rational reaction of the follower. In light of such leader’s decision, the follower selects a feasible solution *x*(*y*) for him aiming to optimize his own objective function; this is, the follower reaction depends on the decision made by the leader. On the other hand, the Nash equilibrium occurs when multiple players simultaneously make a decision at the same level considering the others competitors’ decisions as fixed. Therefore, any player can take into account possible changes regarding the strategies of the others players. Hence, the Nash equilibrium can be appropriately applied to multi-objective programming problems.

For the case of bi-level programming problems, the approach that seems to be more appropriated is finding the Stackelberg equilibrium, which considers the existence of a predefined hierarchy among players. First, the leader makes his decision and based on that decision, the follower chooses its decision and the leader knows exactly the follower's decision. Therefore, the leader has the possibility to take into account the optimal response of the other player. In [14], the authors justify their proposed approach by assuming that a bi-level problem may be modeled as a Nash game if the players try to optimize their own benefit in a non-cooperative way.

However, reducing a bi-level programming solution into the concept of classical Nash equilibrium is not a simple and straightforward issue. In this regard, we refer the reader to [40–42]. First, [41] studied a bi-objective problem, where they compared both the Nash and Stackelberg approaches. In both cases a genetic algorithm was employed, but in the Stackelberg case, the authors define a leader and a follower for each experimental scheme. The experimental results reveal that the Stackelberg approach attains better results (although it is more computationally expensive). They conclude that Nash and Stackelberg frameworks are significantly different and the correct approach depends on the particular problem. Also, [42] adapted multi-objective optimization techniques to solve a particular class of bi-level programming problems; where the optimal bi-level solution is determined by the Pareto optimal points, corresponding to the non-dominated points that belong to the intersection of the two efficiency sets. However, in order to find the efficient points, the cones must be convex and in most cases they are not. Furthermore, the proposed methodology lies in independently solving two bi-objective problems interchanging the leader and follower; this experimentation was conducted in order to validate the relationship between them. They conclude that multi-criteria techniques have not proved to solve bi-level problems. Also, [43] studied the differences between bi-level and bi-objective programming problems. The authors note that although there have been attempts to establish a relationship between both types of problems a formal agreement has not been reached. Furthermore, several counterexamples that refuse any relationship between them could be found in the literature (e.g. [44], [45], [46] and [47]). The authors empirically (and graphically) have shown that optimal solutions of a bi-level problem are not found into the Pareto optimal solution of a bi-objective problem.

## Problem Formulation

A bi-level programming problem is a mathematical programming problem which is composed of an upper-level and a lower-level problem. In this paper, the upper-level problem aims to minimize the connection cost, while the lower-level problem seeks to minimize the average message delay time. When solving the bi-level problem both the decision maker of the upper-level (hereafter referred to as the *leader*) and the decision maker of the lower-level (hereafter referred to as the *follower*) interact to get the best solution. For a formal definition of the Bi-level Local Area Network Design Problem (BLANDP) and based on the model proposed in [14], let *N* = {1,2,…,*n*} be the set of *users* (e.g. routers) in the telecommunication network, and let *G* = (*V*, *E*) be an undirected graph, where *V* = {*v*_1_, *v*_2_,…, *v*_*m*_} is the set of vertices (*clusters*) and *E* = {(*v*_*p*_, *v*_*q*_): *p* < *q*} is the set of edges (*bridges*) that connect the clusters. For each cluster, the maximum traffic capacity *C*_*p*_ that can flow through it is known. Also, for each bridge the average response time *b*_*pq*_ to route a package between the respective clusters is known. We assume that the traffic characteristics between users are known and summarized in the users traffic matrix *S*, where an element *s*_*ij*_ ∈ *S* represents the traffic from user *i* ∈ *N* to user *j* ∈ *N*. Two cost elements are considered in the problem: connection cost between clusters {*w*_*pq*_: (*p*, *q*) ∈ *E*} and connection cost between users and clusters {*α*_*ip*_: *i* ∈ *N*, *p* ∈ *V*}.

Consider the following decision variables:

$$y_{ip}=\{\begin{array}{cc} 1, & \mathrm{if user i is asigned to cluster p}\\ 0, & \mathrm{otherwise} \end{array}$$

$$x_{pq}=\{\begin{array}{cc} 1, & \mathrm{if cluster p is conected to cluster q}\\ 0, & \mathrm{otherwise} \end{array}$$

The decision variable *x*_*pq*_ is defined as *x* ∈ *X*, where *X* is a set of spanning trees. From these decision variables other important elements related to the traffic in the network will be defined. To define these terms the introduction of the following concept is needed. A path between 0 ∈ *V* and *r* ∈ *V*, i.e., *path*(0, *r*), is a sequence of vertex without repetition (*v*_*i*−1_, *v*_*i*_) ∈ *E* for all *i* = 1,…,*r*. A vertex *v*_*k*_ is called intermediate vertex in *path*(0, *r*), if {*v*_0_,…, *v*_*k*_,…, *v*_*r*_}. Similarly, we define the concept of intermediate edge as the set of all edges (*p*, *q*) in *path*(0, *r*). With the above definitions and concepts we precise the following terms, let:

Γ be the total offered traffic in the network, which can be computed as

$$\Gamma =\sum_{i=1}^{N}\sum_{j=1}^{N}s_{ij}\;\text{or by}\;\Gamma =\sum_{p\in V}\sum_{q\in V}t_{pq}$$

T be the traffic matrix between clusters, which can be computed as T = *Y* ^*T*^ *SY*, where *Y* is the clustering matrix, which assigns users to clusters. An element *t*_*pq*_ of this matrix represents the traffic forwarded from cluster *p* ∈ *V* to cluster *q* ∈ *V*.

*L*(*x*)_*k*_ be the total traffic at cluster *k* ∈ *V*, this can be computed as

$$L{\left(x\right)}_{k}=\sum_{p\in V}t_{pk}+\sum_{q\in V\backslash \{\text{k}\}}t_{kq}+\sum_{\left\{p,q\in V|\text{k}\in \text{path}(\text{p},\text{q})\right\}}t_{pq}$$ (1)

*F*(*x*)^(*p*.*q*)^ be the total traffic which flows on the bridge (*p*, *q*) ∈ *E*' ⊆ *E*, computed as

$$F{\left(x\right)}^{\left(p.q\right)}=\sum_{\left\{k,r\in \text{V}|(\text{p},\text{q})\in \text{path}(\text{k},\text{r})\right\}}t_{kr}$$ (2)

The leader's optimization problem consists in determining the best allocation of users to clusters such that the connection costs are minimized. On the other hand, the follower's optimization problem is to determine the subset of edges *E*' ⊆ *E* that while forming a spanning tree T = (*V*, *E*') in *G*, minimize the average message delay time in the network. It should be noted that *L*(*x*)_*k*_ and *F*(*x*)^(*p*.*q*)^ strongly depend on the network’s configuration defined by the spanning tree *x*. The bi-level mathematical model of the considered problem is given by:

*Bi-level Local Area Network Design Problem* (*BLANDP*)

$$\underset{y}{\text{min}}\sum_{(p,q)\in E}w_{pq}x_{pq}+\sum_{i=1}^{n}\sum_{p\in V}\alpha_{ip}y_{ip}$$ (3)

subject to:

$$\sum_{p\in V}y_{ip}=1\;\forall i=\{1,\ldots ,n\}$$ (4)

$$y_{ip}\in \{0,1\}\;\forall i=\{1,\ldots ,n\},p\in V$$ (5)

where *x* solves

$$\underset{x}{\text{min}}\frac{1}{\Gamma }\left[\sum_{p\in V}\frac{L{\left(x\right)}_{p}}{C_{p}-L{\left(x\right)}_{p}}+\sum_{p\in V}\sum_{q\in V}F{\left(x\right)}^{\left(p,q\right)}b_{pq}\right]$$ (6)

subject to:

$$\sum_{(p,q)\in A}x_{pq}=m-1$$ (7)

$$\sum_{\left(p,q)\in (S,S\right)}x_{pq}\leq \left|S\right|-1\;\forall S\subseteq V$$ (8)

$$L{\left(x\right)}_{p}<C_{p}\;\forall p\in V$$ (9)

$$x_{pq}\in \{0,1\}\;\forall p,q\in V$$ (10)

The objective function in Eq (3) minimizes the total connection costs (leader's aim). The first term refers to the connection cost between clusters determined by the spanning tree, while the second term refers to the allocation cost between users and clusters. Eq (4) states that each user can be connected only into a single cluster. The follower's objective function given in (6) minimizes the average message delay time. The total average delay in the LAN is composed of the delays of the clusters and the bridges (see [13]), and as a results it is a nonlinear function. It should be noted that a tree must satisfy the following tree conditions: have *m* − 1 edges, be connected and acyclic. Moreover, any two of these conditions imply the third one. Therefore Eq (7) states that a tree must have exactly *m* − 1 edges, while Eq (8) enforce the constraint that the edges in *T* cannot form cycles, where (*S*, *S*) denotes all edges that go from one vertex in the set *S* to another vertex in the set *S*. Both equations imply a spanning tree. Constraint (9) states a capacity condition for the traffic that flows at a given cluster. Finally, constraints (5) and (10) indicate the binary nature of the decision variables.

In order to assure that the bi-level problem defined by (3)-(10) we assumed that if the follower have multiple optimal responses for any leader’s decision, then the follower’s decision that is more convenient for the leader will be selected. This case is known as the optimistic version of the bi-level problem.

## Solution Algorithm

Bi-level programming problems are generally difficult to solve because evaluation of the upper level objective function requires the solution of the lower level optimization problem. Furthermore, since the lower level considered in this paper consists of a nonlinear constraint, the bi-level problem is inherently a non-convex programming problem. The lower level problem aims to solve a minimum average message delay spanning tree problem at each step of the optimizing process of the upper-level problem, which is basically an assignment problem. Therefore, a Genetic Algorithm (GA) is proposed for solving the problem described above.

The motivation for using a GA in this particular application is based on the fact that it is a very flexible technique which can be adapted in several ways to several optimization problems by suitably defining the criteria used in the operators of the solution procedure. GAs use strategies for diversification maintaining most of the good quality solutions; and have proved to be efficient on solving a variety of multi-objective, robust optimization and bi-level problems. Since GAs are population-based metaheuristics an efficient representation of the solutions in the form of a chromosome is required. Prior of describing the proposed GA implementation, we define the solution coding (chromosome) and the objective function evaluation (fitness function).

### Solution coding and objective functions evaluation

**Fig 1:**
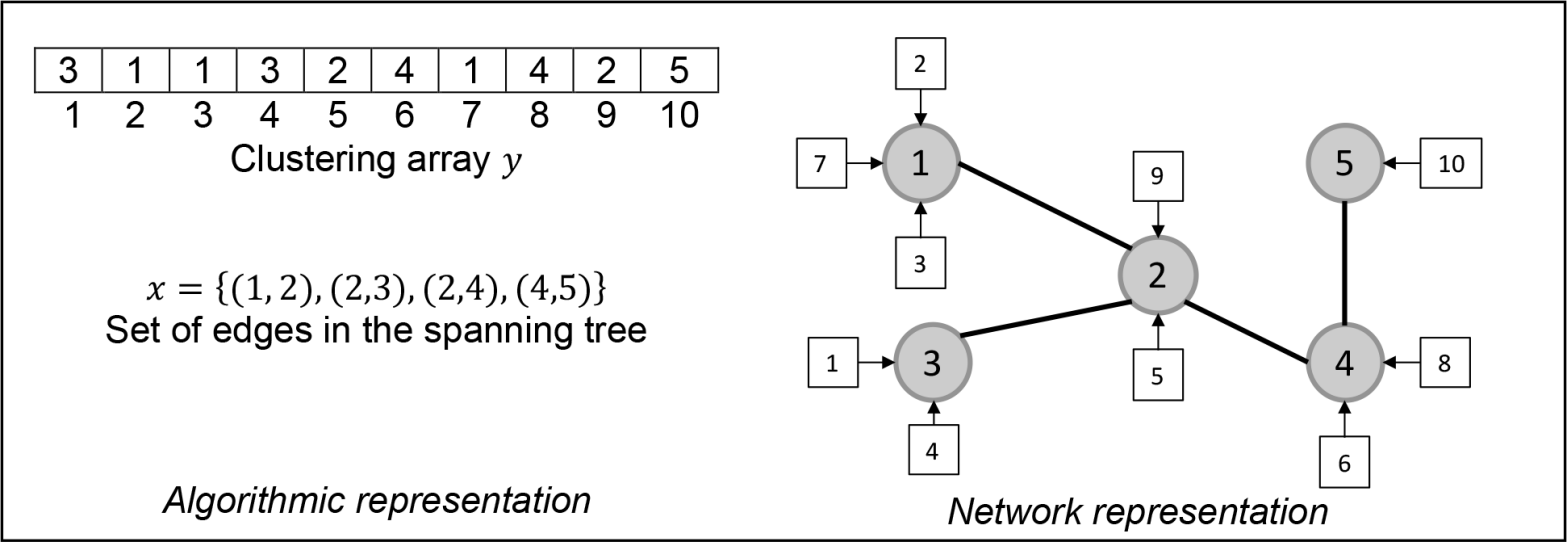
Solution coding.

The purpose of our problem is to group users and assign them to clusters so that the clusters form a spanning tree that does not violate the capacity bridge constraints. Therefore, the simplest structure to represent a feasible solution might be to consider an array *y* = 〈*y*(1), *y*(2),…, *y*(*n*)〉, where *n* is the number of users in the network. Each position of *y* indicates the cluster *p* to which the *i* −th user has been assigned (*y*(*i*) = *p*). A feasible solution must satisfy the requirement that each user must be assigned to only one cluster, which is easily satisfied here. On the other hand, the spanning tree configuration is represented by a list of edges *x* = {(*p*, *q*) | (*p*, *q*) ∈ *E*'} such that *card*(*x*) = *m* − 1. See example in Fig 1.

In Fig 1, it is shown a possible representation of a solution with five clusters and ten users. For instance, *y*(4) = 3 means that user 4 has been assigned to cluster 3. In the graphical representation of the network, the circles represent clusters while the squares represent users. Thick lines represent the bridges linking two clusters, and arrows indicate the assignment of users to clusters. The solution shown is feasible if the cluster capacity constraint is satisfied.

From an algorithmic point of view, a bi-level problem is solved as follows: in an iteration *t* the leader proposes a solution *y* ^*t*^; restricted to that solution, the follower obtains its response *x* ^*^ (*y* ^*t*^) aiming to minimize the lower level function *f*_*L*_(*x* ^*^ (*y* ^*t*^)). This is, the follower rationally reacts to the solution made by the leader and passes its solution to the leader which evaluates the upper level objective function *fu*(*y* ^*t*^, *x* ^*^ (*y* ^*t*^)). Then, if it is necessary the leader changes its decision by proposing a different solution *y* ^*t*+1^. The procedure is repeated until some stopping criterion is met. The difficulty in solving the bi-level programming problem arises from the necessity of solving the lower level at each iteration of an algorithm that handles solutions for the upper level. Hence the computational time consumed for solving the lower level needs to be minimized. Typically, when it is possible, the lower level is optimally solved; in the cases when the lower level problem is NP-hard or a strong combinatorial problem, it is assumed that since the follower rationally reacts to a leader’s decision, an acceptable solution (good quality in a low computational cost) will be made. By considering this approach an acceptable Stackelberg equilibrium is reached.

Now, that bi-level procedure is adapted for the BLANDP in the next manner: given a new assignment in the *y* array, the minimum average message delay spanning tree *x* ^*^ (*y*) is attained by solving a variant of the MST (Minimum-weight Spanning Tree). A greedy constructive algorithm is proposed in order to build feasible solutions for the minimum average message delay spanning tree problem. The constructive method accomplishes its task by adding, at each step, exactly one edge to a current partial solution (i.e. adding a cluster to the current tree). Before describing the algorithms it should be noted that in Eq (1), where the traffic at cluster *k* is computed, only the third term depends of the spanning tree. Therefore, we define the partial traffic at cluster *k* as *L*'_*k*_ = ∑_*p*∈*V*_ *t*_*pk*_ + ∑_*p*∈*V*\{k}_ *t*_*kq*_, and the partial traffic which flows on every pair of bridges as *F*'(*x*)^(*p*,*q*)^ = *t*_*pq*_ + *t*_*qp*_. Also, the partial average message delay caused by the edge *e*_(*p*,*q*)_ ∈ *E* is defined as:

$$Q(e_{\left(p,q\right)})=\frac{1}{\Gamma }\left[\frac{L{'}_{p}}{C_{p}-L{'}_{p}}+\frac{L{'}_{q}}{C_{q}-L{'}_{q}}+F{'}^{\left(p,q\right)}b_{pq}\right]\;\forall (p,q)\in E$$ (11)

The proposed constructive algorithm is an iterative process that is similar to Kruskal's algorithm. At iteration *k*, the algorithm selects an edge *e*_*k*_ = *e*_(*p*,*q*)_ with the minimum partial average message delay value *Q*(*e*_*k*_) from the set of edges which have not been included in the tree, that criterion can be used to augment the current tree while the feasibility is maintained (i.e. that edge whose inclusion would not result in a cycle) and adds it to the current tree T = (*V*, *E* ^*k*−1^ ∪ {*e*_*k*_}) where *T*_*v*_ = {*v* ∈ *V*: *v is in the current tree T*} and *T*_*E*_ = {(*p*, *q*) ∈ *E*: (*p*, *q*) *is in the current tree T*}, note that *T*_*E*_ ⊆ *E*'. The algorithm stops when |*T*_*E*_| = *m*−1. After an edge *e*_(*p*,*q*)_ is added to the tree, the current average message delay is updated with the following formula:

$$Q(e_{\left(p,q\right)},T)=\frac{1}{\Gamma }\left[\sum_{p\in T_{V}}\frac{L'{\left(x\right)}_{p}}{C_{p}-L'{\left(x\right)}_{p}}+\sum_{(p,q)\in T_{E}}F'{\left(x\right)}^{\left(p,q\right)}b_{pq}\right]$$ (12)

Once the tree is constructed, *x* ^*^ (*y*) is obtained and the evaluation of the lower level function *f*_*L*_(*x* ^*^ (*y*)) is made. Then, considering the leader’s decision *y* and rationally follower’s reaction *x* ^*^ (*y*) the cost associated to the upper lever function *f*_*U*_(*y*, *x* ^*^ (*y*)) is computed as follows:

$$f_{U}(y,x^{*}\;(y))=\sum_{(p,q)\in E'}w_{pq}+\sum_{i=1}^{n}\sum_{p\in \{V:y(i)=p\}}\alpha_{ip}y_{ip}$$ (13)

### Genetic algorithm

**Fig 2:**
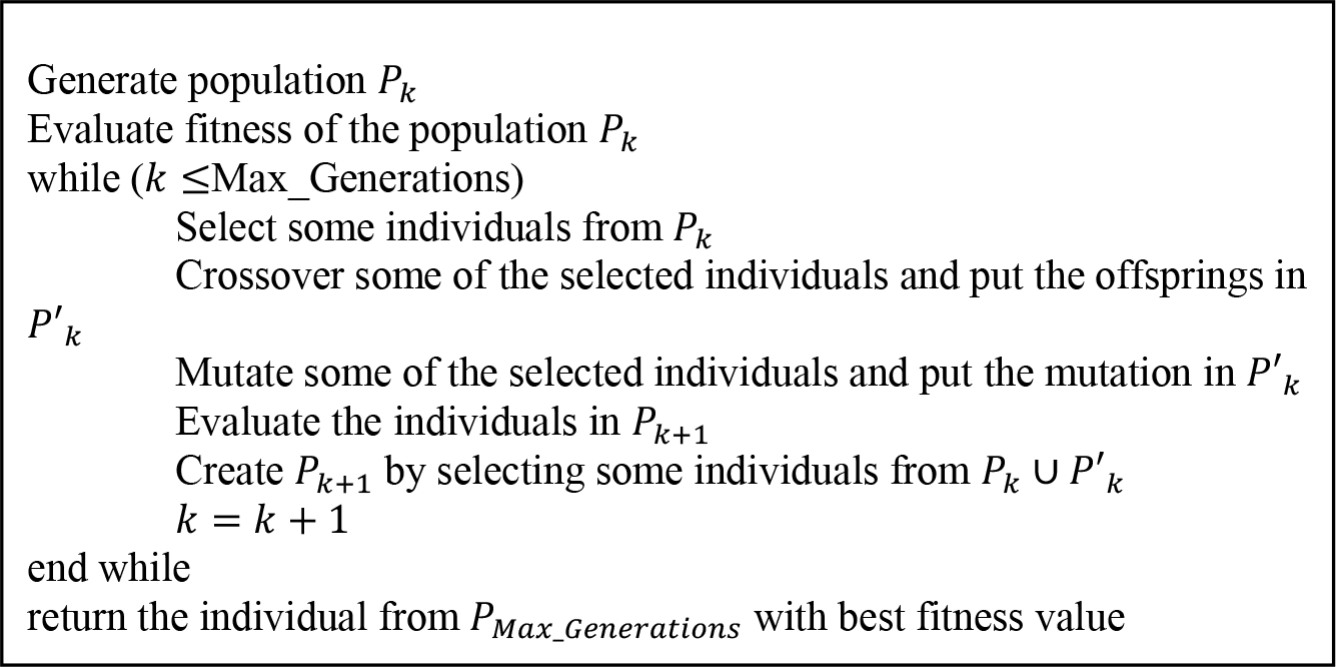
Genetic Algorithm’s framework.

In this subsection the developed GA is described. Genetic algorithms operate on a set of individuals (solutions) which form a population for a determined generation, then either two individuals are selected and combined in a crossover operation or each individual is mutated. These crossover and mutations are randomly performed in order to generate new solutions. Then, based on a selection criterion, the strongest individuals (those with the best value of a performance metric) survive and remain for the next generation. The process is repeated until some stopping conditions are fulfilled. A general framework for GAs is shown in Fig 2. In order to solve the BLANDP, an appropriate adaptation of the different components of the GA needs to be described.

In order to perform the selection of the individuals in the GA a fitness value needs to be defined. This fitness value measures the quality of the individuals and enables them to be compared. Since we are solving a bi-level programming problem the fitness value considered must be the leader’s objective function value, i.e. the value given by formula (13).

Initial population: In order to generate a diverse population the individuals are randomly created. A particular individual *y*_*k*_ is created in the following way: for each of the *n* users a random number between 1 and |*V*|, where |*V*| represents the total number of clusters in the network, is chosen and added to the current individual. This process is repeated until the initial population is complete, i.e. *k* reaches the desired number for the size of the population. It is important to mention that if individuals are created in the described manner, the feasibility is guaranteed.

After the initial population is created, for each individual the follower’s rational reaction *x* ^*^ (*y*) is obtained. Then, the fitness value *f*_*U*_(*y*, *x* ^*^ (*y*)) is evaluated.

Selection: Due to the efficiency for ranking the solutions avoiding premature convergence, a tournament selection strategy is selected to be implemented in the algorithm. A tournament consists in selecting an individual and randomly matches it to another individual of the population, then compare their respective fitness value and identify the winner. The winner will be the individual with the best fitness value, i.e. the individual with lower connection cost. These matches are made for all of the individuals in the current population. In order to allow that the individuals with best fitness value remain in the population, a predefined number of tournaments will be conducted. It is worthy to note that if very few tournaments are done, then the ranking of the individuals tend to have a lot of randomness; on the other hand, if numerous tournaments are done, the ranking will be biased to the better individuals eliminating the diversity required for the GAs. After the population is ranked accordingly to the results obtained from the tournaments an elitist selection is made. In other words, half of the individuals better ranked will enter to the genetic operators.

The algorithm considers two genetic operators: crossover and mutation. Hence, for each of the individuals chosen in the selection phase a random number between 0 and 1 is generated. If the random number is less or equal than a predefined parameter the individual will enter to the crossover operator; otherwise, it will enter to the mutation one.

Crossover: This is the main genetic operator, so the probability to enter in this phase is greater than 0.50. The crossover simulates the reproduction between two individuals, called the parents. The procedure is as follows: the current individual is randomly matched with another individual from the population (where population means the complete population not only the half corresponding with the selected individuals). Then, both parents are combined in order to produce two offsprings. A standard single crossover point is implemented; such point is randomly selected for the first parent (P1) and also considered for the second parent (P2). One of the offsprings will inherit the first part of P1 and the second part of P2; the other offspring will be created in the opposite way.

Mutation: In the case when an individual had entered in this phase a small change in its codification occurs. This random change will gradually incorporate new characteristics to the population which allows exploring new regions of the solution space. Since the crossover produces offsprings with the same characteristics than the parents, the mutation takes an important place in the algorithm in order to have diverse individuals. The mutation is performed by selecting a component of the current solution and randomly change it for another number between 1 and |*V*|; i.e. an specific user is allocated to another cluster.

**Fig 3:**
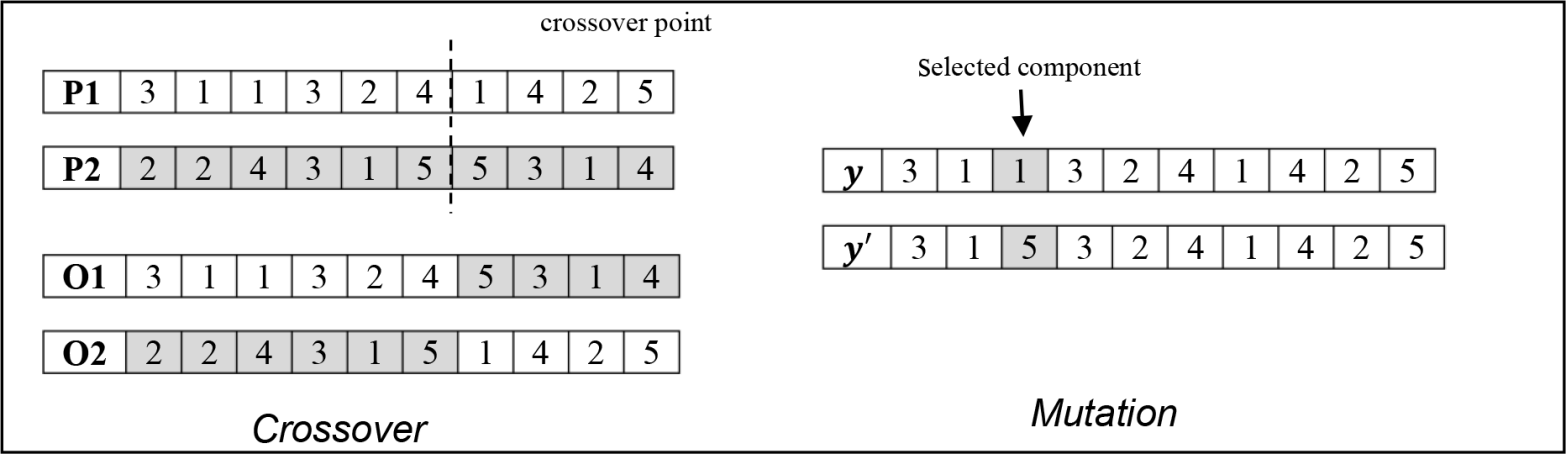
Genetic operators.

An illustration of the considered genetics operators is shown in Fig 3. It is important to mention that crossover and mutation ensure feasibility of the new created individuals and for each of the new solutions the rational reaction of the lower level needs to be computed again.

## Computational Experiments

The computational testing can be divided in three main parts. First, we used the set of three instances reported in [14] as benchmark. In this set of instances the users in the network vary from 8 to 50, and the clusters vary from 4 to 10. Second, a discussion regarding the solution reached by the Nash-Genetic algorithm in the instances existing in the literature is presented. Finally, a new set of 10 instances was created varying in size. The new set of instances varies in size from 60 to 300 users and from 15 to 50 clusters in the network. The parameter tuning for the benchmark and the generated instances was conducted and it is showed in the corresponding section. All instances considered in our experimentation are available upon request. Both set of instances are used to analyze the performance of the solution method developed in this paper. All the experimentation conducted in this paper was implemented in C++ using the Microsoft Visual Studio 2010 programming environment via the Windows 7 operative system. The experiments were conducted on an HP Compaq 6000 Pro PC with a Pentium Dual-Core processor at 3.00 GHz and 2.00 GB RAM.

### Experimentation on benchmark instances

As it is mentioned above, in the literature only exist three instances for this problem. However, the instances are incomplete because some parameters are described without presenting the exact data, this is, the probability distribution for the parameters is indicated. Hence, the missing information was generated with the same procedure reported in [48] and [14]. The tested problems are specified in Table 2.

**Table 2:** Data for the benchmark instances.

	Benchmark 1	Benchmark 2	Benchmark 3
Number of users	8	30	50
Number of clusters	4	6	10
Clusters’ connection cost (*w*_*pq*_)	*w*_*pq*_ ∼ U(100,250)		
Users’ connection cost (*α*_*ip*_)	*α*_*ip*_ ∼ U(1,100)		
Capacity (*C*_*p*_)	50	300	500
Clusters’ response time (*b*_*pq*_)	0.1	0.1	0.1

Before conducting the numerical experimentation, the tuning for the parameters involved in the Genetic algorithm is done. The main parameters are: the number of generations (*G*), the size of the population (*P*), the probability (*π*) of entering into the crossover or the mutation phase and the number of tournaments in the selection phase. The latter is set to 5 because it is a recommendable and common value in order to maintain a balance between diversification and intensification of the solutions. For the other three parameters, i.e. *G*, *P* and *π*, preliminary numerical tests are used to set the values of the required parameters. The preliminary tests consist on conducting runs with different settings of the parameters and then making numerical comparisons.

**Fig 4:**
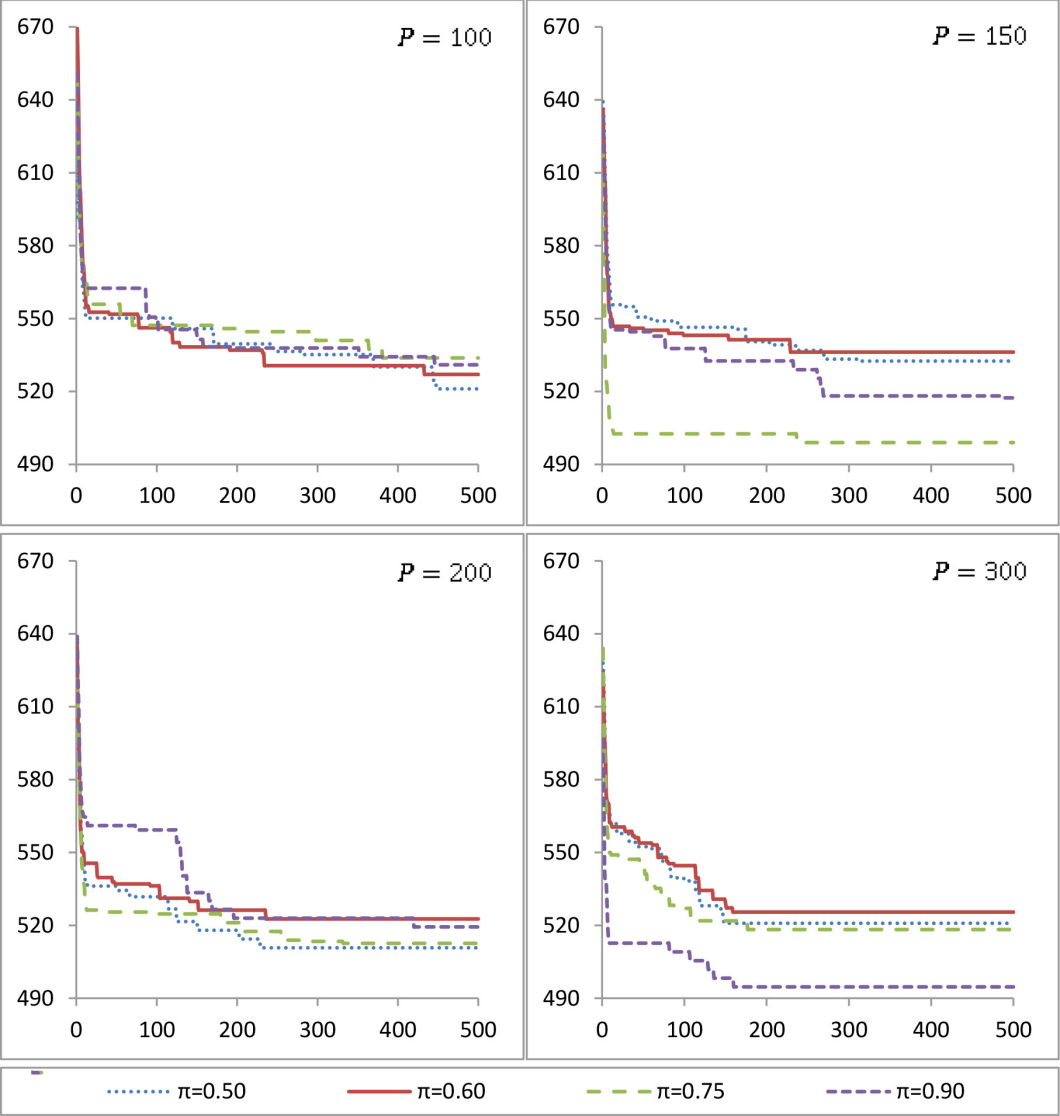
Parameter tuning for Benchmark 1.

First, based on the size of the instances we established four different possible values for *P*, those are 100, 150, 200 and 300. Then, we did the same for *π* selecting 0.50, 0.60, 0.75 and 0.90. The value of *G* was set as 200, 300, 400 and 500 for the preliminary tests. As an example, the numerical results for Benchmark 1 are plotted in Fig 4. This part of the experimental work was carried out to analyze the behavior of each proposed combination of parameters in the three considered instances. Due to the stochasticity presented in the methodology, for each one of the different combination of parameters the genetic algorithm was run ten times.

In order to select the parameters for the genetic algorithm, a full factorial design was conducted. The design consists in three treatments and four levels with 10 replications. Two response variables are considered, the leader’s objective function and the required time. The results of the experimental design showed that the three factors have significant effect in both response variables. A maximum value for the required time was considered for discarding some combinations of the treatments’ levels.

On the other hand, the results obtained from the computational tests described in the full factorial design were graphically analyzed. In Fig 4 the average of the values obtained after the ten runs of each of the parameters combinations for Benchmark 1 are plotted. It is important to mention that for all the instances the same methodology was followed but in this paper we only show the results for Benchmark 1 as an illustration. The axes correspond to the number of generations and the leader’s objective function value. The results from varying the genetic operators probability (*π*) can be seen in each of the plots. Also, one plot corresponds to a different size of the population (*P*).

When comparing the different parameters combinations from Fig 4, it can be observed that their efficiency is quite similar as far as solution quality and solution time is concerned (the time is directly related with the number of generations). It seems difficult to identify some variants clearly dominating others. Therefore, considering that computing the rational reaction of the follower is not straightforward but difficult due to its complexity, a smaller population is desired. On the other hand, an intermediate value in the number of generations seems to be a good one taking into consideration that long runs will incur in higher computational time. Also, we can identify some critical points where the quality of the solution would not improve any more.

After having analyzed the results obtained from the design of experiments and supported by the graphical illustration, the parameters setting for Benchmark 1 is *π* = 0.75, *P* = 150 and *G* = 300. It can be seen from Fig 4 that, when higher probability *π* was considered, the algorithm reached better leader’s objective function values. Considering that the same leader’s solution may obtain different follower’s reaction, the probability of entering to the crossover or mutation operators is *π* = 0.75. Also, as it is mentioned above, due to the size of the population *P* negatively affects to the required time, then a smaller value of *P* is preferred, i.e. *P* = 150. Finally, the number of generations also has an impact in the required time, so 300 generations seemed to be an efficient value based on computational time and leader’s objective function value. For example, the average time consumed for the 500 generations of the ten runs of each configuration was 4.5, 6.7, 9 and 13.7 seconds for 100, 150, 200 and 300 individuals in the population, respectively. The parameters setting is presented on Table 3.

**Table 3:** Parameter setting for the benchmark instances.

	Benchmark 1	Benchmark 2	Benchmark 3
Genetic operators probability (*π*)	0.75	0.50	0.60
Size of the population (*P*)	150	200	200
Number of generations (*G*)	300	400	500

After have tuned the parameters for each benchmark instance, 50 runs of the code were performed in order to assess the quality of the proposed genetic algorithm. In Table 4 the results from the computational experimentation are shown. The “Best” column represents the best value obtained from the 50 runs of each problem. In the “Average” column the average of the 50 runs is indicated and in the “Worst” column the higher obtained cost is indicated. Then, the “Gap” column is calculated as $\text{Gap}=\frac{\left|best-Average\right|}{best}\times 100\%$. The sample standard deviation is presented in the “Std. Dev.” column. The “# Best” and “% Best” columns indicate the total number of times and the percentage of times when the best value was reached, respectively. Finally, the “Time” column indicates the average time (in seconds) for solving 50 times each problem.

**Table 4:** Numerical results for the benchmark instances.

	Best	Average	Worst	Gap	Std. Dev.	# Best	% Best	Time
**Benchmark 1**	493	498.94	502	1.20	4.31	27	54	4.230
**Benchmark 2**	1203	1226.30	1282	1.94	18.04	22	44	13.951
**Benchmark 3**	1602	1652.89	1738	3.18	38.60	18	36	54.877

From Table 4 it can be observed that for Benchmark 1 the algorithm reached the best value in more than half of the 50 runs. Moreover, the average from all the runs is very near from the best obtained value and the standard deviation indicates that the values are around the average; the small gap obtained (1.20%) confirms the good performance of the developed algorithm in this problem. The consumed average time is 4.23 seconds.

The results for Benchmark 2 indicate that despite the expected increase in the computational time (almost 14 seconds), the algorithm reached a very good gap between the best obtained value and the average of the 50 runs. This gap is lower than 2%. Also, the best value was obtained in almost the half of the experimentation (in 22 of the runs). Finally, the numerical experimentation conducted for Benchmark 3 was not as good as the previous ones but the results are still reasonable. The best value was reached in 18 of the 50 runs, while the gap increased to 3.18% and the consumed time was of 54.9 seconds. These results were clearly affected by the difficulty of finding the follower’s rational reaction.

### A comparison between the Stackelberg-Genetic and the Nash-Genetic algorithms

In this subsection, the solutions obtained by the Genetic algorithm developed in this paper (SG, hereafter) and by the Nash-Genetic algorithm (NG, hereafter) proposed in [14] are discussed.

The solutions considered for the SG are the ones presented in subsection 4.1. On the other hand, for obtaining the solutions from the NG we emulated the algorithm described in [14] in order to solve the BLANDP. Next, a brief general description of the NG is shown. Let (*y* | x) be the string representing the potential solution for a bi-objective optimization problem. Then *y* denotes the subset of variables controlled by the leader and optimized accordingly to the connection costs. Similarly *x* denotes the subset of variables controlled by the follower and optimized with respect to the average delay in the network. According to the Nash perspective, the leader optimizes (*y* | x) with respect to his objective function by modifying *y* while *x* is fixed by the follower, i.e. the leader will find *y* ^*^ (*x*). Symmetrically, the follower optimizes (*y* | x) with respect to his objective function by selecting *x* while *y* is fixed by the follower, i.e. the follower will find *x* ^*^ (*y*). In the same way that in [14], two different populations are considered; the first one is named *pop*1 corresponds to the assignments *y* associated with the connection of the users to clusters and the second one is named *pop*1 corresponds to the spanning trees *x* resulting from connecting the clusters. In each population the fitness of the individuals is evaluated with its corresponding objective function, i.e. the leader or follower’s objectives.

**Fig 5:**
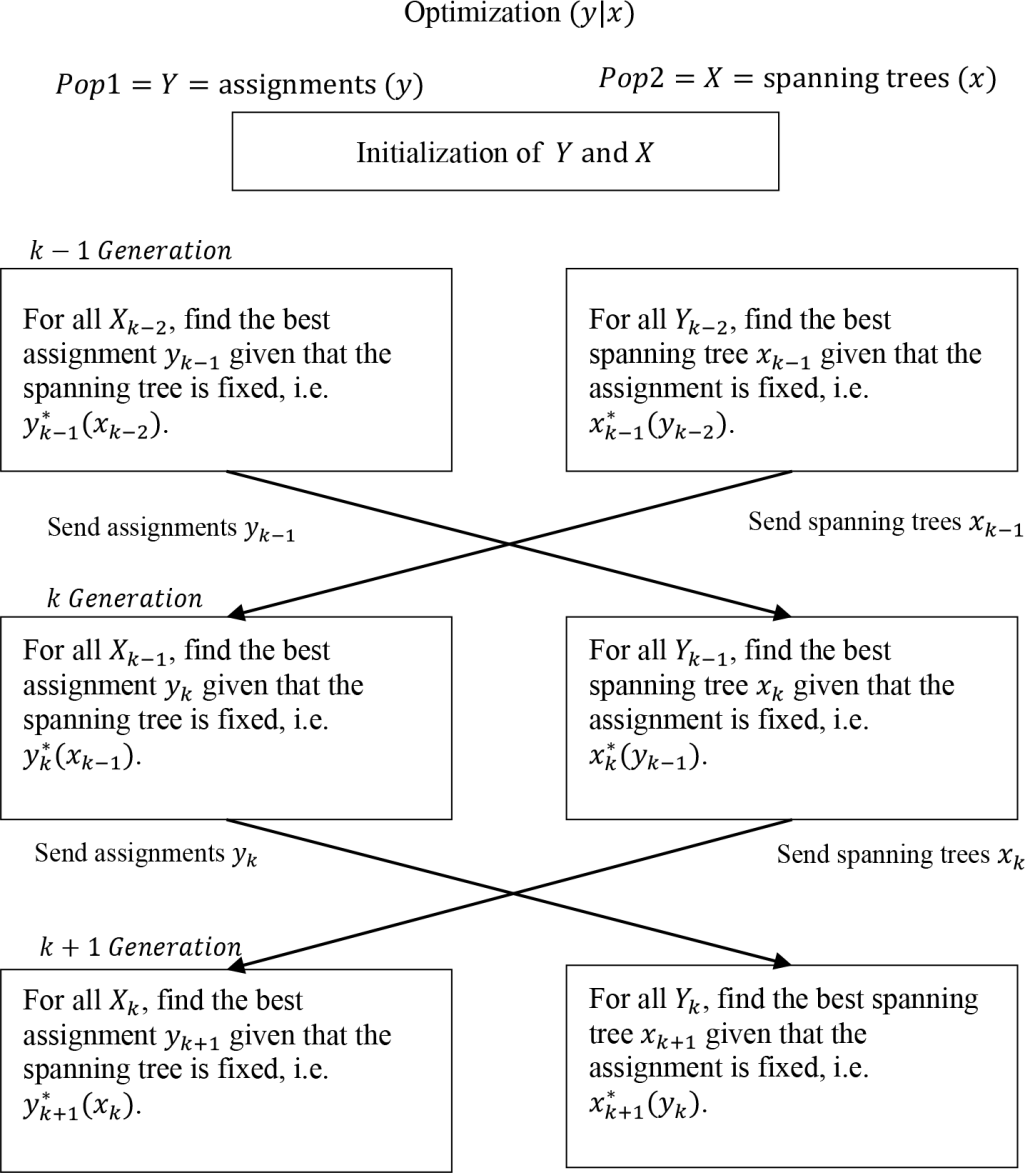
The process of the emulated Nash-Genetic algorithm is shown.

Let *y*_*k*−1_ be the best value found by the leader at generation *k*−1 and *x*_*k*−1_ be the best value found by the follower at generation *k*−1. At generation *k*, the leader optimizes *y*_*k*_ while using *x*_*k*−1_ in order to evaluate (*y* | x). In the same way, the follower optimizes *x*_*k*_ while using *y*_*k*−1_ in order to evaluate (*y* | x). After the optimization process, the leader sends the best value *y*_*k*_ to the follower who will use it at generation *k* + 1; then the follower makes the same procedure. The Nash equilibrium is reached when neither the leader nor the follower can further improve their criteria without affect the other party interests. This procedure is illustrated in Fig 5.

It is important to mention that for the Nash-Genetic algorithm that we implemented the genetic operators (crossover and mutation) and the selection phase are the same that the ones described in the third section.

In order to show the performance of the NG, we solved the benchmark instances and compared the obtained results. Each instance was run 50 times, as in the SG algorithm. Let (*pop*1, *pop*2) denote the selected size for the leader’s and follower’s populations, respectively. For Benchmark 1, the populations are (10,10) due to the fact that 16 spanning trees are possible. For both problems, Benchmark 2 and Benchmark 3 the populations are (100,100). The algorithm stops when the best individual for both populations is the same, this is, when no improve in at least one objective function can be made. The values for both, SG and NG algorithms are presented in Table 5. The description of the columns is the same than the one for Table 4.

**Table 5:** Numerical results for the comparison between SG and NG.

	SG				NG			
	**Best**	**Average**	**Gap**	**Time**	**Best**	**Average**	**Gap**	**Time**
**Benchmark 1**	493	498.94	1.20	4.230	635	635.00	0.00	0.751
**Benchmark 2**	1203	1226.30	1.94	13.951	1217	1234.74	1.44	27.655
**Benchmark 3**	1602	1652.89	3.18	54.877	1518	1716.82	11.58	170.469

The main comments about the values shown in Table 5 are concerned with the computational time and with the leader’s objective function. When the instance contains a number of clusters that demands an evolutionary process in the follower’s population, the required time for the NG is increased. This is clearly caused by the existence of two populations, which require entering to the genetic operators for evolving.

Furthermore, there is no evidence for indicating whether the leader’s objective function value increases or decreases. However, it is very important to note that we could find Pareto-efficient solutions that lead us to better objective function values, but in most of the times these solutions are not going to be in the inducible region of the bi-level problem. As it was mentioned in subsection of related literature, the optimal bi-level solution is not necessarily found in the set of efficient solutions of a bi-objective problem. Then, we cannot make a valid comparison about the objective function reached by SG and NG algorithms since the NG solutions will not be bi-level feasible ones (in general).

The important issue here is to show the significant difference in solving a bi-level programming problem without properly consider the leader and follower roles. Also, the main detail in considering the NG approach is that obtaining *y* ^*^ (*x*) in the follower’s population may not be adequate for solving bi-level problems since finding the best assignment for a particular spanning tree does not concord with hierarchy considered in bi-level programming. Nash-Genetic algorithms seem to fit better for solving multi-objective problems.

### Robustness of the SG algorithm

The objective of this section is to show that the performance of the algorithm is steady and efficient. In order to do this, a new set of 10 larger-size instances was randomly generated maintaining the same structure than the benchmark instances. We keep considering the user’s and cluster’s connection cost as *α*_*ip*_ ∼ U(1,100) and *w*_*pq*_ ∼ U(100,250), respectively. The cluster’s response time is standardized as 0.1 for all the instances. The rest of the data for each instance is given in Table 6.

**Table 6:** Data for the generated instances.

Instance	Users	Clusters	Capacity
GI-1	60	15	600
GI-2	70	20	750
GI-3	80	20	1000
GI-4	90	25	1000
GI-5	100	25	1500
GI-6	150	30	2500
GI-7	200	30	4000
GI-8	250	40	5000
GI-9	300	20	6500
GI-10	100	50	1200

Since the second set of instances contains larger size problems, more different possible values were considered for each parameter. This is, for *P* we considered 100, 200, 300, 400 and 500. Similarly for *π* we tested 0.50, 0.60, 0.70, 0.80 and 0.90. The number of generations *G* was set to 500, 1000, 1500 and 2000, in order to select the more appropriate value for each instance. Preliminary testing was conducted in the same manner than for the benchmark instances considered. An analogous full factorial design of experiments was conducted. Also, the results were supported by the corresponding plots in the same manner than Fig 4. Then, the appropriate parameters setting is presented on Table 7.

**Table 7:** Parameter setting for the generated instances.

Instance	*π*	*P*	*G*
GI-1	0.70	100	500
GI-2	0.70	200	500
GI-3	0.80	200	500
GI-4	0.70	300	1000
GI-5	0.60	300	1000
GI-6	0.60	400	1000
GI-7	0.50	400	1500
GI-8	0.50	500	1500
GI-9	0.60	300	1000
GI-10	0.50	500	1500

Then, in Table 8 the numerical results from the computational experimentation considering the parameters described in Table 7 are presented. The headings of Table 8 are analogous to Table 4. In the same manner than for the benchmark set of instances, 50 runs were conducted for the set of generated instances.

**Table 8:** Numerical results for the generated instances.

Instance	Best	Average	Worst	Gap	Std. Dev.	# Best	% Best	Time
GI-1	2773	2927.62	3272	5.58	165.12	14	28	66.711
GI-2	3838	3940.85	4457	2.68	175.38	20	40	82.532
GI-3	3745	4018.69	4645	7.31	321.93	12	24	85.726
GI-4	4728	4972.73	5589	5.17	303.82	16	32	105.248
GI-5	5397	5662.83	6227	4.92	307.00	10	20	111.392
GI-6	7369	7891.67	9167	7.09	693.39	21	42	163.966
GI-7	9529	10353.87	11534	8.65	711.07	9	18	206.473
GI-8	14669	15189.88	16025	3.55	533.99	6	12	289.007
GI-9	12902	13236.83	14726	2.60	470.31	16	32	151.057
GI-10	10043	10484.02	11376	4.39	332.24	3	6	178.264

From Table 8 it can be appreciated that the Stackelberg genetic algorithm has a steady performance. The gap between the best leader’s objective function value reached and the average from the 50 runs is less than 9% for all the instances. The standard deviation is small in 8 of the 10 instances; this indicates that in most of the runs the algorithm converges to a region that contains good quality solutions. The percentage of times that the algorithm repeats the best obtained solution is acceptable, from 18% to 42% of the runs. When the number of clusters increases, such as, in GI-8 and GI-10, the algorithm decreases its performance reaching the best value only in 6 and 3 runs, respectively. This behavior is due to the significant increase in the follower’s decision space; and since the lower level solution’s method is an efficient heuristic, larger variability appears. Increasing in the required time was expected, since the size of the generated instances augmented. However, the required time seems to have a polynomial increase. It is mainly affected by the number of generations and in a lower way by the size of the population.

Finally, it is worth to remark that an increase in the number of clusters will exponentially augment the number of possible trees (follower’s decision space). The well-known Cayley’s formula can be applied for computing the total number of feasible trees associated with the follower’s decision. Hence, the algorithm’s performance is negatively affected by this fact. The heuristic considered for finding the follower’s rational reaction is a main topic for further research.

## Conclusions and Further Research

In this paper a bi-level programming model for analyzing a local network design problem was proposed. In this problem, the leader decides the allocation of users to clusters in order to minimize the connection costs; while the follower connects the clusters forming the spanning tree that minimizes the average network delay. For efficiently solving this problem, a genetic algorithm considering an acceptable Stackelberg equilibrium was proposed. This algorithm deals with the fact the lower level problem cannot be optimally solved in a straightforward manner, hence the follower’s rational reaction need to be defined. In order to solve the lower level, we implemented a heuristic procedure that seemed to be efficient in sense of quality and required time.

Numerical results were conducted taking as a basis benchmark instances in the literature for this problem and a new set of randomly generated instances. The conclusions we can make after having analyzed the results are that the best leader’s objective function value was found several times and the computational consumed time is very acceptable for a problem from this nature. The robustness of the proposed algorithm is showed by numerical experimentation conducted to different size instances. The performance of the algorithm is stable without having much variation due to the different instances’ components.

It is important to mention that, because no efficient algorithm for bi-level optimization associated with large-scale network problems is available, an iterative optimization-assignment algorithm has usually been used in network design problems (e.g., traffic signal setting and expansions of link capacities). This algorithm consists of iterating between the upper-level optimization problem with fixed lower-level decision variable values, and lower-level optimization problem (average traffic delay time) with fixed upper-level decision variable values. However, it is demonstrated theoretically and empirically that this iterative algorithm does not necessarily converge to the exact solutions of Stackelberg games, but is rather an exact and efficient algorithm for solving Cournot-Nash games, in which each player attempts to maximize his/her utility or payoff noncooperatively and assumes that his actions will have no effect on the actions of the other players (see [49] and [50]). Here, it should be particularly mentioned that the iterative optimization-assignment algorithm presented in [14] obviously does not solve the BLANDP considered in this paper because the optimal solution of upper-level problem is the target one if the lower-level decision variable values are fixed. We can appreciate this fact by looking at the discussion of the results presented in subsection 4.2.

As an area of opportunity, we identified that since the difficulty immersed in dealing with the hard-combinatorial follower’s problem an alternative methodology needs to be proposed. In this paper, a heuristic method was implemented for finding a follower’s rational reaction. This methodology could give us different follower’s responses for the same leader’s solution but not necessarily the best spanning tree. This issue affected the efficiency of the genetic algorithm in the experimentation associated with the larger-size instances. Therefore, a methodology that considers a pool of spanning trees and the evaluation of them in order to obtain the best follower’s response for each leader’s decision seems to be a good option. It is evident that this methodology could be very expensive in sense of computational time, so the use of parallel computing it is necessary. Moreover, this alternative may lead us to design a co-evolutionary algorithm where both populations improve in an independently fashion but always considering the existing hierarchy, i.e. for each leader’s decision find the best follower’s response in the corresponding evolved population.
